# Fabrication of Micro-Punch Array by Plasma Printing for Micro-Embossing into Copper Substrates

**DOI:** 10.3390/ma12162640

**Published:** 2019-08-19

**Authors:** Tomomi Shiratori, Tatsuhiko Aizawa, Yasuo Saito, Kuniaki Dohda

**Affiliations:** 1 Komatsuseiki Kosakusho, Co. Ltd., R&D section, Suwa, Nagano 392-0012, Japan; 2 Surface Engineering Design Laboratory, Shibaura Institute of Technology, Ohta, Tokyo 144-0045, Japan; 3 Chuo Denshi Kogyo, Co. Ltd., Ujyou, Kumamoto 869-0512, Japan; 4 Department of Mechanical Engineering, Northwestern University, Evanston, IL 60208, USA

**Keywords:** packaging, copper substrate, micro-embossing, micro-textures, plasma printing, micro-punch array, screen printing, AISI316

## Abstract

Copper substrates were wrought to have micro-grooves for packaging by micro-stamping with use of a AISI316 stainless steel micro-punch array. The micro-texture of this arrayed punch was first tailored and compiled into CAD data. A screen film was prepared to have the tailored micro-pattern in correspondence to the CAD data. A negative pattern to this screen was printed directly onto the AISI316 die substrate. This substrate was plasma nitrided at 673 K for 14.4 ks. The unprinted die surfaces were selectively nitrogen super-saturated to have sufficiently high corrosion toughness and hardness; other surfaces were masked by the prints. The two-dimensional micro-pattern on the screen was transformed into a three-dimensional nitrogen supersaturated micro-texture embedded in the AISI316 die. The printed surfaces were selectively sand-blasted to fabricate the micro-textured punch array for micro-embossing. A uniaxial compression testing machine was utilized to describe the micro-embossing behavior in copper substrates and to investigate how the micro-texture on the die was transcribed to the copper. The micro-punch array in this study consisted of three closed loop heads with a width of 75 µm and a height of 120 µm after plasma nitriding and sand-blasting. Since the nitrogen supersaturated heads had sufficient hardness against the blasting media, the printed parts of AISI316 die were removed. The micro-embossing process was described by comparison of the geometric configurations between the multi-punch array and the embossed copper plate.

## 1. Introduction

A key technology in the plastic mold packaging of hollowed GaN high electron mobility transistor (HEMT) chips lies in micro-joining with sufficient interfacial integrity between the copper substrate and the plastic molds [1]. In particular, liquid crystal plastic (LCP) molds have been utilized for packaging with sufficient gross-leak proof [2,3,4]. The copper substrates must have micro-textures for those plastic molds to be joined with sufficiently high integrity of the interface strength between the LCP molds and copper substrate.

Authors have proposed a non-traditional method to fabricate the micro-punch with the use of low temperature plasma nitriding [5]. This plasma printing is based on the principle that a two-dimensional micro-pattern is transformed into the three-dimensional nitrogen-embedded micro-structure to be working as the micro-punch array heads [6]. The initial micro-pattern was directly printed onto the stainless steel die substrate by ink-jet printing [7], screen printing [8], and maskless lithography [9].

In the present study, a micro-punch array was designed to have multi-heads with a geometric configuration of three continuous closed loops. Tailored CAD data were first transferred to screen film for printing as a negative micro-pattern to these loops. This negative micro-pattern was screen-printed onto an austenitic stainless steel AISI316 die substrate. This die substrate was plasma nitrided at 673 K for 14.4 ks to develop the two-dimensional micro-pattern into a three-dimensional nitrogen supersaturated microstructure in the die. A micro-punch array was fabricated by mechanically removing the printed parts of the AISI316 die. Scanning electron microscopy (SEM), electron diffractive X-ray spectroscopy (EDX), and a non-contact three-dimensional measuring device were utilized to describe the evolution of the nitriding-induced microstructure by element mapping and the dimensional change, respectively. A compression testing machine was utilized to emboss the micro-punch array into the copper substrate for micro-texturing.

## 2. Experimental Procedure

**Figure 1:**
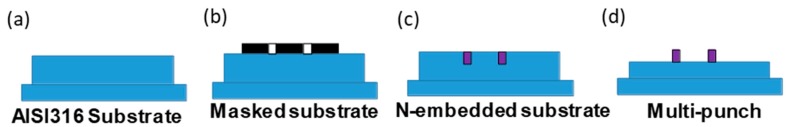
Plasma printing procedure in the present study. (**a**) The starting AISI316 substrate, (**b**) masked substrate by screen printing, (**c**) nitrogen-embedded substrate by plasma nitriding, and (**d**) multi-punch formed by blasting.

An AISI316 die substrate with dimensions measuring 24 mm × 12 mm × 5 mm was utilized as a substrate material. Its surface was mirror-polished for plasma printing. The average roughness (*Ra*) of the AISI316 die substrate was 0.010 μm. The plasma printing procedure was composed of the screen printing, low-temperature plasma nitriding, blasting, and micro-embossing using a tensile tester. The present plasma printing process consisted of three steps, as illustrated in Figure 1. First, the negative micro-pattern of arrayed punch heads was printed as a two-dimensional mask onto a mirror-polished AISI316 substrate (Figure 1a). Figure 1b depicts the screen-printed mask pattern. Second, this printed substrate was plasma nitrided at 673 K for 14.4 ks to selectively super-saturate nitrogen onto the unprinted substrate surfaces (Figure 1c). The printed surfaces were not nitrided and maintained the same hardness as the matrix so that they could be mechanically removed with ease from the substrate to form the multi-punch array, as shown in Figure 1d.

### 2.1. Screen Printing onto Die Substrate

**Figure 2:**
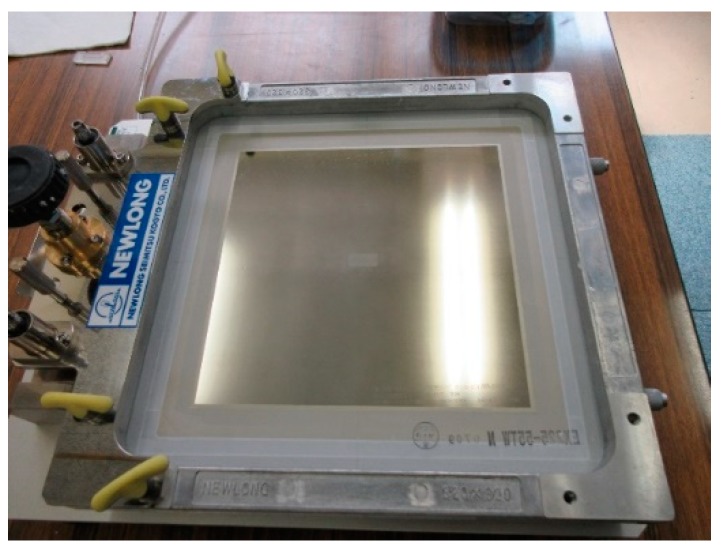
Appearance of screen-printing system.

**Figure 3:**
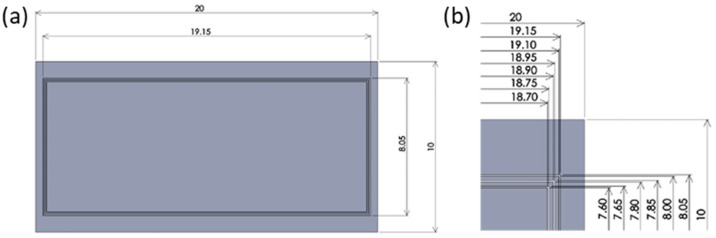
Geometry of a closed loop patterns on the CAD data. (**a**) General pattern of three closed loop, (**b**) details of closed loop patterns.

The screen-printing system (NEWLONG, Co., Ltd., Tokyo, Japan), as shown in Figure 2, was employed to print the CAD-designed micro-pattern onto the substrate’s surface. The three closed loop patterns with a width of 50 µm were directly printed onto the AISI316 die surface within the range of 19.15 mm × 8.05 mm, as depicted in Figure 3a. The details of the CAD data for three closed loops are shown in Figure 3b. These loops were aligned to have mutual distances of 150 mm between adjacent loops. In the following experiments, a screen with three closed loops was employed to print its negative pattern onto the surface of the AISI316 die. Ink for screen-printing must be optimally selected from among several candidates to have sufficient thermal resistance during plasma nitriding (at 673 K). A polymer-based ink has the risk to diminish itself during plasma nitriding at 673 K. In practical operations, specially formulated TiO_2_ ink (Teikoku Printing Inks Mfg. Co., Ltd., Tokyo, Japan), without the use of thinning agents, was used for directly printing onto the die surface and then dried at 373 K for 1.2 ks in air.

### 2.2. Low-Temperature Plasma Nitriding

**Figure 4:**
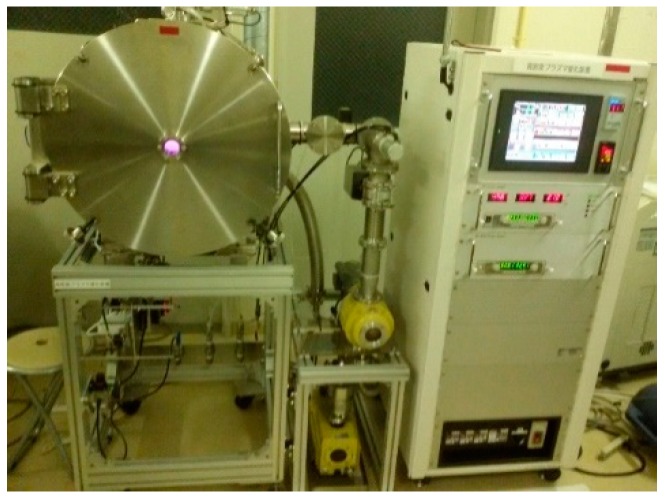
Appearance of the plasma nitriding machine.

A high-density RF (radio frequency)/DC (direct current) plasma nitriding system (YS-Electric Industry, Co., Ltd., Yamanashi, Japan) was utilized to selectively super-saturate nitrogen on the unprinted substrate surfaces at 673 K for 14.4 ks at 70 Pa, as shown in Figure 4. After evacuation down to 0.1 Pa, the nitrogen gas was introduced to pre-sputter the printed die surface for 1 ks under a DC-bias of −600 V. After re-evacuation, the specimen was heated to 673 K under a nitrogen atmosphere at 250 Pa. Then, the nitrogen hydrogen mixture gas was introduced with a flow rate of 160 mL/min for the nitrogen and 30 mL/min for the hydrogen, respectively. After plasma nitriding, the specimen was cooled in the chamber under a nitrogen atmosphere. The micro-printed AISI316 substrate surface was fully covered by a plasma sheath with a high nitrogen ion and NH-radical densities; enough to drive the nitrogen super-saturation at lower temperatures [5]. This selective anisotropic nitrogen-embedding process resulted in selective hardening and selective nitrogen concentrations. The printed surface remained as a matrix hardness, while the unprinted surfaces were selectively hardened to 1400 HV for the AISI316 substrates, as reported in Reference [10].

### 2.3. Mechanical Blasting Process

**Figure 5:**
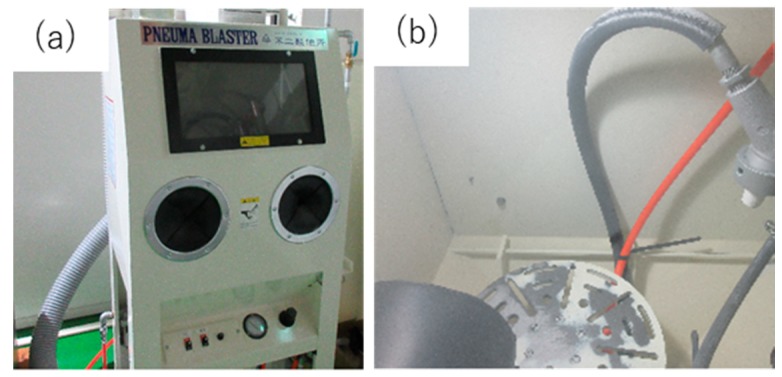
Mechanical blasting equipment. (**a**) Overall image of blasting equipment and (**b**) the shooting stage for blasting the substrates in manual operation.

Mechanical blasting equipment (Fuji Manufacturing Co., Ltd., Tokyo, Japan) was also employed to selectively remove the un-nitrided parts and the masked ink from the substrate. Owing to the hardness distribution, the nitrided areas were left as a punch head while the un-nitrided areas were completely removed by this processing. Figure 5 depicts the blasting apparatus for manual operation. The blasting rate was controlled by the shooting speed of the blasting media. The punch height was also varied by duration time. In the following blasting step, fine silica particles with an average diameter of 5 μm were utilized as the blasting medium. The shooting rate was maintained constant at 2 m/s and the shooting angle was 60 degrees. As depicted in Figure 5b, the specimen was fixed into a jig on the shooting stage for continuous shooting operation. The duration time was selected to be 300 s in the experiments. According to Reference [11], the punch height reached 120 μm by blasting the printed parts of substrate for 300 s.

### 2.4. Micro-Embossing Process

**Figure 6:**
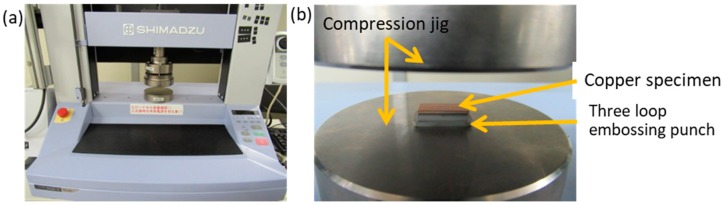
Micro-embossing in the uniaxial compression onto the copper plates. (**a**) Overview of the compression testing machine and (**b**) enlarged view of the micro-embossing experiment condition.

A precision universal testing machine AUTOGRAPH AGS-X 10 kN (SHIMADZU Corporation, Kyoto, Japan) was utilized for micro-embossing, as shown in Figure 6a. The plasma-printed punch array was set onto a compression test jig and embossed into the copper specimen, as shown in Figure 6b. The compression testing conditions were as follows: The compressive velocity was constant at 0.1 mm/s until the applied load reached maximum at 10 kN and the duration time was set at 10 s. An oxygen-free copper plate (20 mm × 10 mm × 1 mm) was employed as a work material for this micro-embossing. The average roughness was 0.093 μm.

### 2.5. Evaluation Method for Micro-Embossed AISI316 Die Substrate

The AISI316 die substrate in each step of the plasma printing process was evaluated by a three-dimensional measurement machine (Alicona Imajing GmbH., Graz, Austria), as well as scanning electron microscopy (SEM; JSDM-IT300LV, JEOL Ltd., Tokyo, Japan). Energy dispersive X-ray spectroscopy (EDX; Pegasus, EDAX, Inc., Tokyo, Japan) was utilized for fine element mapping.

## 3. Experimental Results

The plasma printing procedure in Figure 1 was put into practice to shape the micro-punch array with three continuous closed loop heads for micro-embossing. A selective nitrogen embedding process using low temperature plasma nitriding was described as an essential step for plasma printing. Secondly, the micro-punch array with three continuous closed loop heads was fabricated using sand-blasting. This micro-punch was embossed into copper plates to demonstrate that plasma printing should work as an effective tool to make micro-textures in copper substrates for packaging.

### 3.1. Selective Nitrogen Embedding

**Figure 7:**
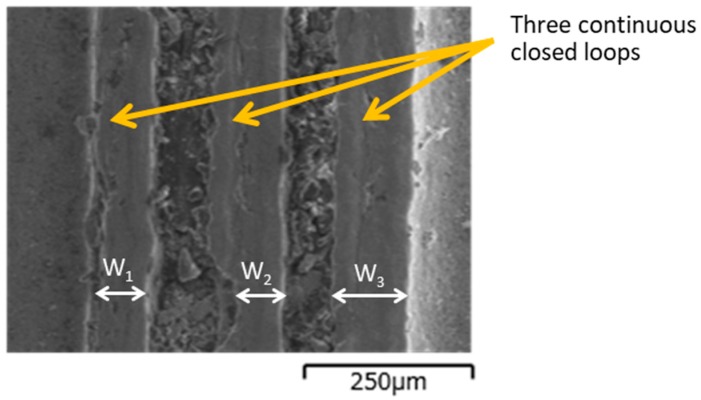
SEM image of the plasma nitrided AISI316 die at 673 K for 14.4 ks before removal of the masks. Three continuous closed loops were seen as three lines and the average width of lines from W_1_ to W_3_ is 90 μm.

The micro-patterned AISI316 die was plasma nitrided at 673 K for 14.4 ks at 70 Pa to demonstrate that nitrogen solutes were homogeneously embedded in the unprinted areas. Figure 7 shows a SEM image of the nitrided AISI316 die before removing the printed masks from the die surface. Three continuous closed loops were seen on the AISI316 surface and were metallic and shining while other areas were still covered by masks. The average width of the three closed loops from W_1_ to W_3_ was 90 μm. There were three closed loop width differences between the CAD-designed 50 µm and the demonstrated 90 µm. This phenomenon suggested that the polymer-based ink was diminished during plasma nitriding.

**Figure 8:**
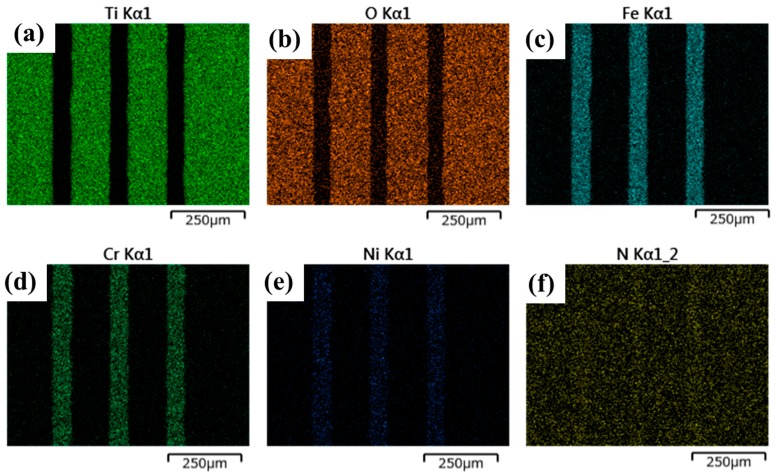
Element mapping analyzed by SEM–energy dispersive X-ray spectroscopy (EDX) for the nitrided AISI316 substrate. (**a**) Titanium, (**b**) oxygen, (**c**) iron, (**d**) chromium, (**e**) nickel, and (**f**) nitrogen.

SEM–EDX was utilized to describe element mapping on the nitrided AISI316. As shown in Figure 8, both titanium (Figure 8a) and oxygen (Figure 8b) are present on the AISI316 die surface, except for on the three lines. The surface other than the three lines were still covered by the TiO_2_ ink. While the iron (Figure 8c), the chromium (Figure 8d), and nickel (Figure 8e) were only detected on the three lined areas. That is, the three lines were a part of the bare AISI316 matrix without TiO_2_ masks. Nitrogen (Figure 8f) was uniformly detected on the whole AISI316 die surface. This proves that the masked AISI316 die was uniformly covered by the plasma sheath and homogeneously nitrided under high nitrogen ion density conditions in plasmas.

### 3.2. Fabrication of Micro-Punch Array

**Figure 9:**
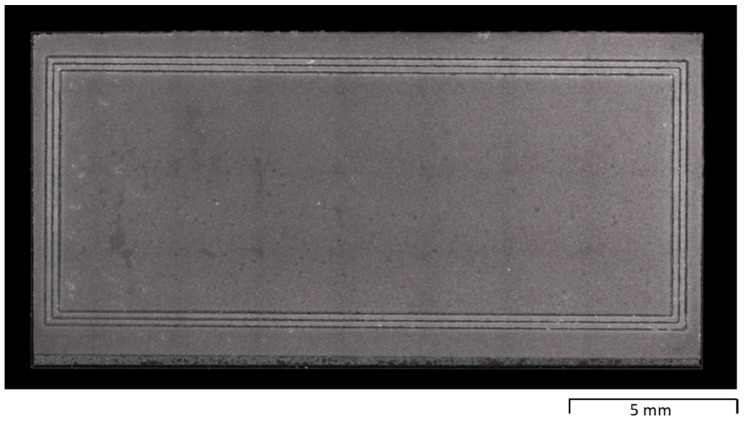
Micro-textured AISI316 micro-punch array with three continuous closed loop heads on the surface.

**Figure 10:**
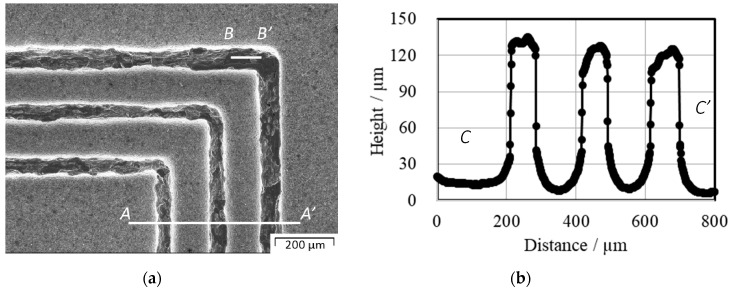
Enlarged SEM image and height distribution of the multi-punch array. (**a**) Enlarged SEM image of micro-textured AISI316 micro-punch array, (**b**) height distribution of the multi-punch array measured along the line *A–A’*. The average punch width at 90 µm height position is 75 µm and the average punch height is 117 μm.

Sand-blasting was utilized to mechanically remove the unnitrided parts from the AISI316 surfaces, as well as the TiO_2_ masks. Figure 9 depicts the micro-punch array with three continuous closed loop heads. Figure 10 shows an enlarged SEM image and height distributions. The height distribution and surface roughness distribution of the multi-punch array was measured at line *A–A’* and line *B–B’*. From Figure 10b, the average height of three closed loop punch reached to the 117 µm. These punches have sharp edge shoulders. The average punch width reached 75 μm at the height position in Figure 10b. The surface roughness in line *B–B’* with the length of 100 μm, was measured by using the cutoff value 20 µm. After JIS B 0601, it reached *Ra* 0.035 µm.

**Figure 11:**
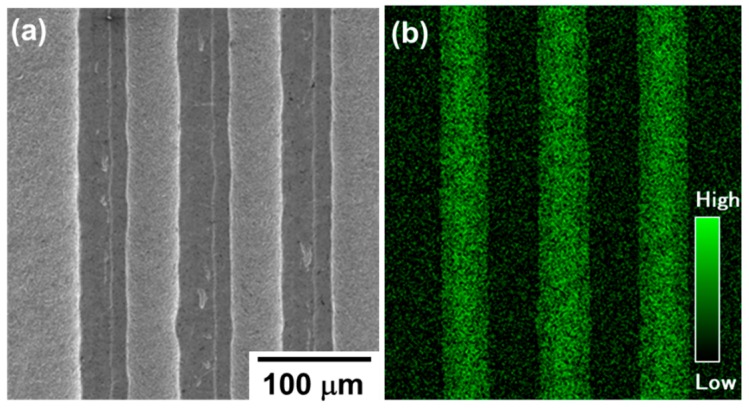
SEM image and nitrogen mapping on the micro-punch heads with three continuous closed loops. (**a**) SEM image and (**b**) nitrogen mapping.

The whole masked and unnitrided AISI 316 parts were removed to leave the three loop heads. Figure 11 depicts an SEM image of three loop punch heads and their nitrogen mapping. AISI316 surfaces were removed into the depth by the sand-blasting. This is because the unprinted surfaces were selectively nitrogen-embedded up to a nitrogen content of 4 to 5 mass% and solid-solution hardened not to be mechanically blasted.

### 3.3. Micro-Embossing into Copper Substrates

**Figure 12:**
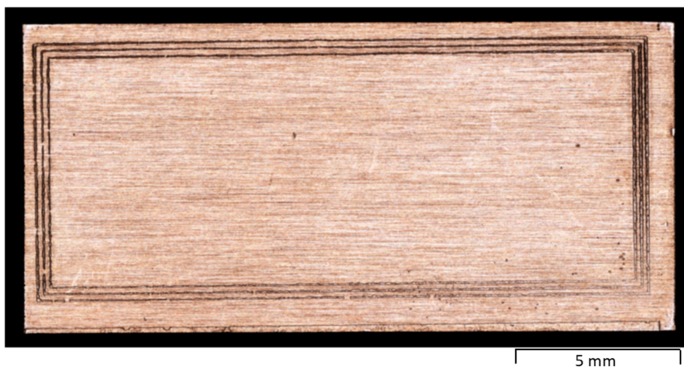
Oxygen-free copper substrate for thermal spreading in the package of hollowed GaN chips after micro-embossing.

The micro-punch in Figure 9 was utilized for micro-embossing process by a Uniaxial compression testing machine. Figure 12 shows the micro-embossed oxygen-free copper substrate for thermal spreading in the package of hollowed GaN chips. Three continuous closed grooves were formed in the copper substrate to surround its center part for packaging the hollowed GaN chips. Three continuous loop punch heads corresponded to the three closed loop grooves.

**Figure 13:**
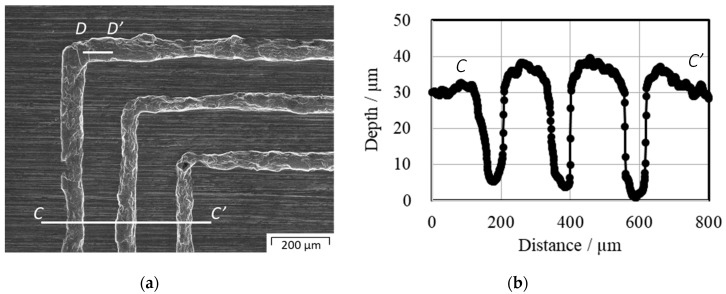
Enlarged SEM image and depth distribution of the micro-embossed oxygen-free copper substrate. (**a**) Enlarged SEM image of micro-embossed oxygen-free copper substrate, (**b**) depth distribution of the micro-embossed oxygen-free copper substrate measured along the line *C–C’*. The average micro-emboss width at a depth of 20 µm is 58 µm and the average micro-embossing depth is 35 μm.

Figure 13a shows an SEM image of the three loop grooves at the corner of the copper substrate. Figure 13b depicts the surface profile of the cross-section along the *C–C’* in Figure 13a. The average width of the micro grooves was 58 µm at a measurement depth of 20 µm and a depth of the micro grooves of 35 µm. Three continuous closed loop heads with a line width of 75 µm and height of 117 µm formed the three continuous micro-grooves, with a width of 58 µm and depth of 35 µm through this micro-embossing process at a 10-kN compression load. The difference the between line widths of the three continuous closed loop heads and the embossed three continuous micro-grooves was not considered due to elastic deformation. The surface roughness in line *D–D’ with the length of* 100 µm, reached Ra = 0.126 mm by using the cutoff value of 20 µm after JIS B 0601 reached *Ra* 0.126 µm.

## 4. Discussion

A micro-milling process was employed to compare the processing time for the fabrication of the same three closed loop AISI316 punch array as that made by the present plasma printing process. A milling tool with a diameter of 10 μm was prepared to achieve fine corner curvatures of the micro-cavities in Figure 3 and Figure 10b. The average machining speed, cutting depth, as well as cutting distance of a single cutting layer were assumed to be 5 mm/s, 5 μm, and 8 μm, respectively, without fracture of the thin milling tools. The milling time to remove the three closed loop areas of 18 mm × 6.5 mm × 0.1 mm and 19.5 mm × 6.5 mm × 0.1 mm was 7 h. In addition, a conventional punch needs heat treatment and required 8 h after the milling process. Including the takt time taken to prepare the computer aided machining (CAM) data for micro-milling, the practical takt time was nearly 2 h. The present plasma printing required only 10 min at most for screen printing, 5 h for plasma nitriding, including heating and cooling, and 5 min for set-up and blasting. No CAM data were necessary since the CAD data were reflected on the screen. This comparison proves the superiority of plasma printing for fabrication of micro-punch arrays for precision mechanical milling processes.

Picosecond laser machining, as well as fiber–laser machining, have been utilized for the formation of micro-groove textures in each copper plate [12]. On the other hand, a micro-grooved copper substrate was fabricated by micro-embossing the plasma-printed punch. The same multi-arrayed punch, was repeatedly utilized for a series of stamping operations to yield the demanded number of copper substrates for packaging. This takt time was reduced to 10 s, including the setting and stamping durations.

The takt time for the production of the micro-textured copper with comparison to the picosecond laser machining to make a micro-grooved copper plate was as follows. In laser micromachining with a beam spot diameter of 50 μm and repetitive frequency higher than 10 MHz [13], the takt time per path for machining a single line down to a depth of 5 μm was only 1 s, including the on/off operation and beam positioning control. Assuming that no adjustment is needed to form the sharp corners of two crossing micro-grooves, the takt time is estimated to be: (1 s) × (12 lines for three micro-grooves) × (7 paths by 35 μm/5 μm) × (double paths for formation of groove side surfaces) = 168 s. Total takt time to complete three closed micro-grooves into the copper substrate was reduced by 1/17 by using the present stamping approach.

Microgroove textures for joining must be tailored to have geometric compatibility to the substrate size and chip allocation on the substrate. When using laser machining, more takt time is necessary to prepare for CAM data and for actual machining operations. The present plasma printing process has the intrinsic flexibility to transcribe a tailored micropattern to the multi-punch array on the die unit for micro-embossing the microgroove textures onto the copper substrate without increasing the takt time of production. Furthermore, this nitrided multi-punch array has sufficient hardness to prolong the die life in practical micro-embossing operations.

## 5. Conclusions

Plasma printing was successfully applied to fabricate a micro-punch array with three continuous closed loop heads. Three loops with a line width of 75 μm and a height of 117 μm were accurately micro-embossed into an oxygen-free copper substrate to form three continuous closed micro-grooves. These microgrooves work as a key-wedge for micro-joining between the copper substrate and plastic molds in packaging. This plasma printing can be utilized to fabricate tailored three-dimensional micro-textures and to improve interfacial integrity in packaging.

This plasma printing has sufficient flexibility to form any complex-shaped micro-groove network into copper-alloy base substrates for plastic packaging. Different from precision mechanical milling, there is no increase in takt time since the plasma printing procedure is indifferent to the complexity of a micro-grooving network. No CAM data are necessary to transform the geometric model in CAD to a three-dimensional punch array. The dimensional accuracy, as well as geometrical topology, are preserved by screen printing and the selective nitrogen supersaturation. Micro-embossing to form the micro-groove network in the substrate, with use of CNC (Computer Numerically Controlled) stamping, requires much less takt time than needed for short-pulse laser machining to each substrate. In particular, the present process is favored for mass production of substrates for plastic mold packaging.
